# Geminate consonant grapheme-colour synaesthesia (ideaesthesia)

**DOI:** 10.1186/s12883-015-0372-7

**Published:** 2015-07-15

**Authors:** Donald F. Weaver, Cassandra L. A. Hawco

**Affiliations:** Toronto Western Research Institute, 60 Leonard Avenue, Toronto, ON M5T 2S8 Canada; University Health Network, 101 College Street, Toronto, ON M5G 1 L7 Canada; Department of Medicine, Division of Neurology, University of Toronto, Suite RFE 3-805, 200 Elizabeth Street, Toronto, ON M5G 2C4 Canada

**Keywords:** Synaesthesia, Ideaesthesia, Double letter perception

## Abstract

**Background:**

Synaesthesia is a neurological condition which manifests clinically as an involuntary experience of a sensory or cognitive pathway upon stimulation of a second unrelated sensory or cognitive pathway

**Case presentation:**

We report a 55 year old male who presented with a life-long history of grapheme-colour synaesthesia in which the triggering grapheme was the double letter ‘ll’ (a geminate consonant), but not ‘l’ as a single letter. This patient’s synaesthesia was also font specific (becoming more evident with serif fonts) and influenced by migraine headache (being suppressed during the prodrome and aura of a migraine headache)

**Conclusion:**

These results suggest that geminate consonants are uniquely processed rather than treated as two individual consonants. Also, the existence of a mechanistic relationship between synesthetic and migrainous events sequence was verified.

## Background

Synaesthesia is a neurological condition which manifests clinically as an involuntary experience of a sensory or cognitive pathway upon stimulation of a second unrelated sensory or cognitive pathway [1–3]. Although there are many clinical expressions of synaesthesia, the most commonly investigated form is an association linking graphemes with colours, termed grapheme-colour synaesthesia [4, 5]. Thus, grapheme-colour synaesthesia is a neurological condition comprising a sensation of colour which occurs simultaneously with viewing graphemes (letters or numbers); the association between a grapheme and a colour is specific, with graphemes corresponding to a specific colour that is largely unchanged throughout the synaesthete’s life [6–8].

We report a patient with grapheme-colour synaesthesia in which the triggering grapheme is the double letter “ll” (geminate consonant), but not “l” as a single letter. In addition to this unique observable, this patient’s synaesthesia is also font-specific (becoming more evident with serif fonts) and influenced by migraine headache (being suppressed during the prodromal and aura phases of a classical migraine headache).

## Case presentation

A 55 year old right-handed male presented with a life-long history of grapheme-colour synaesthesia in which the double letters “ll” (*e.g.* in the words “will”, “silly”) are both equally perceived in a vivid blue colour. When the letter “l” is presented singly (*e.g.* “colour”), it is not seen as blue; when a word contains two “l” letters separated by another letter (*e.g.* lily), neither “l” is seen as blue. He describes the colour blue as being on the page, rather than existing “in his mind’s eye”. No other letters or numerals are seen in colour. He also reported no other forms of synaesthesia and to the best of his knowledge there was no family history of synesthesia. The experience has been invariant his entire life. Apart from a lifelong problem with right-left confusion (first noted by a school teacher when the patient was age five years), he reported no other neurological symptoms; apart from migraine headache, he has no neurological conditions or disorders and is not taking any medications. His neurological examination is entirely normal and neural imaging (CT scan) did not indicate any abnormalities.

The patient has a history of classic migraine with aura since age 15 years. Five-six times per year, he experiences a full spectrum prodrome → aura → pain phase migraine event. The prodrome onsets two hours to two days prior to the aura; prodromal symptoms include altered mood, food craving, irritability and odour sensitivity. The aura occurs 60–80 min prior to the pain phase and typically involves a scintillating scotoma, comprising partial alteration of this visual field with coloured zigzag lines. The pain phase involves a pulsatile, left-sided unilateral pain associated with nausea and rarely vomiting. Significantly, the prodrome and aura phases of this migraine spectrum are associated with suspension of the grapheme-colour synaesthesia. During this time, he sees the double letters “ll” in a normal black colour, the same as all other letters. Indeed, he acknowledges that the suspension of his “ll” grapheme-colour synaesthesia is a foreshadowing indicator for the onset of a migraine within the ensuing day.

Synaesthetic experiences were evaluated using a computerized test adapted from Simner and co-workers [9, 10]; 36 graphemes (i.e. 26 letters and digits 0–9) were presented using an electronic palette of 13 colours based on Berlin and Kay’s colour terms (black, dark blue, brown, dark green, gray, pink, purple, orange, red, white, light blue, light green and yellow) [11]. Graphemes were displayed in Ariel font against a white background. The subject was seated comfortably at a blank table; the subject sat 85 cm from a LCD monitor with a 60 Hz refresh rate.

The patient reports a font-specific influence upon his synaesthesia, as has been previously described by Ramachandran and Hubbard [2]. For sans-serif fonts (Arial, Calibri, Helvetica) the “ll” grapheme is seen with light blue shadows; for serif fonts (Garamond, Georgia, Times New Roman) both of the “l”s are seen entirely in a more vivid darker blue colour. This effect could be modified by embedding the “ll” grapheme as a serif font within a longer word given in a sans-serif font. For example, if the word “intellectualization” is presented in an Arial font, but with the “ll” embedded in a Times New Roman font, then the “ll” grapheme is seen with blue shadowing, rather than entirely in a blue colour. Conversely, when “intellectualization” is presented with a Times New Roman font, with the “ll” grapheme in Arial, then the “ll” appears entirely in a darker blue.

The “ll”-blue synaesthesia in this patient is shown to be context-dependent and directed by the derived meaning of a stimulus. When the “ll” grapheme is inserted in words or pseudowords, it is always perceived as blue; however, when the “ll” grapheme was inserted into numerical arrays, the blue colour is not perceived. On a single page, a 36x10 array of 5–8 character long words, pseudowords or numbers was created (120 of each). When the “ll” grapheme was inserted into a number (*e.g.* 34ll927), the blue colour was not seen. Conversely, when the number eleven was inserted into a word (lu11aby) or pseudoword (sn11up) it was frequently seen in blue, with an 86 % synesthetic response for words and a 67 % response for pseudowords. Also, the presence of the “ll”-blue synaesthesia, enables the patient to identify the “ll” geminate (double) consonant more efficiently in words and pseudowords than other geminate consonants. Using single page 36x10 arrays of 5–8 digit words and pseudowords, the patient was 2.4-fold slower in identifying instances of the “ss” geminate consonant in comparison with the “ll” geminate consonant. All of these 36x10 array tests were performed reproducibly at one month intervals over a three month period with the exact same shades of blue being reported each time.

To assess the dynamic limits of his grapheme-colour synaesthetic phenomenon, we exposed the patient to presentations in which the “ll” grapheme rotated smoothly, or morphed into other characters, or disappeared abruptly. Rotating the letters and morphing them into another character both caused an abrupt change in colour due to the change or failure in letter identification. Finally, when the “ll” grapheme was embedded in words and pseudowords, but presented in colours other than black (*e.g.* red, green, yellow) he was still able to perceive the blue coloration, but was slower to recognize it; this was most marked when the “ll” grapheme was presented in yellow.

## Conclusions

Grapheme-colour synaesthesia is the most frequently studied form of synaesthesia, but is always reported with single letters or single numbers as the inducers for the synesthetic colour response [12, 13]. This unique case report describes geminate consonant grapheme-colour synaesthesia in which double letter (*not* single letter) stimulation is correlated with perception of the colour blue. Although not previously reported in synaesthesia, the uniqueness of double letter perception in neurological conditions has been previously recognized. Miceli et al. [14] reported a patient’s selective difficulty in spelling words that contain geminate consonants. The likelihood of geminate consonant deletion was significantly greater than a deletion of a consonant in a non-geminate cluster while there was no difference in the likelihood of a substitution of both geminate consonants compared to substitution of only one consonant in a non-geminate cluster. Also, substitution of a single geminate consonant was unlikely to occur, significantly lower than the probability of substitution of only one consonant from a non-geminate cluster [14]. These results suggest that geminate consonants are uniquely processed and are not merely treated as two individual consonants in sequence. Tainturier and Caramazza [15] in studying a case of acquired dysgraphia similarly described graphemic representations not to be simple sequences of graphemes, but rather complex structures that independently encode information in a system in which double letters hold a special status.

In addition to the uniqueness of the double letter inducer of the synesthetic response, this case has two other unique characteristics. First, the synesthetic response is suppressed during the prodromal and aura phases of migraine. Alstadhaug and Benjaminsen [16] have reported a case in which migraine elicited synaesthesia in a person who was otherwise not a synaesthete; although the case reported in our study is the converse to the one presented by Alstadhaug and Benjaminsen, it does verify the existence of a mechanistic relationship between synesthetic and migrainous events. (Possibly the ischaemic vasoconstriction arising from the cortical spreading depression of Leão, implicated in the pathogenesis of migraine prodrome and aura, is mechanistically able to either activate or deactivate the synaesthesia-producing cortical cross-activation [17, 18]. Second, the patient presented in our case report demonstrates font-specific synesthetic responses. Although not previously extensively investigated in synaesthesia research, there are suggestions that different fonts may undergo different neural processing and that different fonts may “prime” the brain differently, producing different emotional and perceptual responses [19, 20].

In addition to synaesthesia, our patient reported chronic problems with right-left confusion; this anecdotal association has been previously documented by Cytowic [21]. Our patient also exhibited a delayed synesthetic response when the “ll” geminate consonant inducer was presented in different colours, most markedly in yellow. This supports the previous research of Nikolić et al. [22] in which the early stages of visual processing are proposed to be involved in the occurence of realistic synaesthetic experiences, also further demonstrating the neural representation of synaesthetic colours closely resembles that of real colours.

Finally, recently is has been suggested that the term synaesthesia may not be correct when applied to the phenomenon of grapheme-colour synaesthesia [23]. Some authors have postulated that the term ideasthesia is a more accurate description of the observed phenomenon rather than synaesthesia [23]. Ideasthesia is a phenomenon in which the activation of a concept (inducer) evokes a perception-like experience (concurrent) [23]. In synaesthesia, both the inducer and the concurrent are sensory, whereas in ideaesthesia the inducer is semantic while the concurrent is sensory. The context-dependent nature of this patient’s synaesthesia, for example its relation to font, demonstrates an inducer as being a concept, rather than sensory input. Therefore, within this definitional context, grapheme-colour synaesthesia would be an ideaesthesia.

### Consent

Written informed consent was obtained from the patient for the publication of this Case report. A copy of the written consent is available for review by the Editor of this journal.
